# NUMB in Endometrial Pathology: From Adenomyosis Expression Patterns to Endometrial Cancer Survival Implications

**DOI:** 10.3390/cimb47121027

**Published:** 2025-12-10

**Authors:** Walid Shaalan, Nourhan Hassan, Mohamed Gamal Ibrahim, Benedikt Schäfgen, Kathrin Haßdenteufel, Julia Gallwas, Ludwig Kiesel, Andreas N. Schüring, Martin Götte

**Affiliations:** 1 Department of Gynecology and Obstetrics, Münster University Hospital, 48149 Münster, Germany; nyehia@sci.cu.edu.eg (N.H.); ibrahim@teamkinderwunsch.de (M.G.I.); benedikt.schaefgen@ukmuenster.de (B.S.); ludwig.kiesel@ukmuenster.de (L.K.); schuering.18@outlook.de (A.N.S.); martin.goette@ukmuenster.de (M.G.); 2 Department of Gynecology and Obstetrics, Heidelberg University Hospital, Im Neuenheimer Feld 440, 69120 Heidelberg, Germany; kathrin.hassdenteufel@med.uni-heidelberg.de; 3 Biotechnology Department, Faculty of Science, Cairo University, Giza 12613, Egypt; 4 Team Kinderwunsch Oldenburg GbR MVZ, 26121 Oldenburg, Germany; 5 Department of Gynecology and Obstetrics, Göttingen University Hospital, Robert-Koch-Street 40, 37075 Göttingen, Germany; julia.gallwas@med.uni-goettingen.de; 6 Cells in Motion Interfaculty Centre, University of Münster, 48149 Münster, Germany

**Keywords:** adenomyosis, Numb protein, endometrial stem cells, myometrium, immunohistochemistry, pathogenesis, TCGA analysis, protein-protein interactions, survival analysis, notch signaling

## Abstract

Adenomyosis, a prevalent gynecologic condition involving invasion of endometrial tissue into the myometrium, remains poorly understood in terms of its molecular pathogenesis. Numb, an important regulator of cellular destiny and stem cell maintenance, has been implicated in numerous proliferative diseases; however, the role for Numb in adenomyosis has not yet been investigated. This research examines the degree to which Numb protein may play a role in the pathogenesis of adenomyosis and additional invasive endometrial diseases. This study analyzed Numb protein expression in tissues from 21 adenomyosis patients and 14 controls using immunohistochemistry. Numb levels were evaluated in eutopic endometrium, ectopic lesions, and myometrium. Additionally, comprehensive bioinformatics analyses were performed including TCGA expression analysis, STRING protein–protein interaction network analysis, and Kaplan–Meier survival analysis to elucidate the clinical significance and mechanistic role of NUMB in endometrial pathology linked to invasive growth. Compared to controls (*p* < 0.001), adenomyosis patients’ eutopic endometrium and myometrium showed considerably higher levels of numb expression. It was predominantly observed in single cells rather than clusters. No significant variation was noted across menstrual cycle phases. Elevated Numb levels in the myometrium suggest a potential role in tissue invasion. TCGA analysis revealed significant associations between NUMB alterations and expression patterns in endometrial carcinoma, with copy number alterations showing a dose-dependent relationship with the expression levels. STRING network analysis identified NUMB as a central hub protein with 20 high-confidence interactions, particularly enriched in Notch signaling pathway components (FDR = 1.0 × 10^−15^). Kaplan–Meier survival analysis demonstrated that endometrial carcinoma patients with NUMB alterations had significantly better overall survival compared to unaltered cases (*p* = 9.119 × 10^−3^), with 92% vs. 73% survival at 100 months. This study presents new evidence of elevated Numb expression in adenomyosis, suggesting that it may play a part in the pathophysiology of the disease and that it is a useful marker for dysregulation of endometrial stem cells. The comprehensive bioinformatics analyses establish NUMB as a central regulator of endometrial homeostasis with significant prognostic value in endometrial malignancies, providing mechanistic insights into the pathogenesis of invasive endometrial diseases and identifying potential therapeutic targets.

## 1. Introduction

Adenomyosis is a benign gynecological condition characterized by the presence of ectopic endometrial glands and stroma within the myometrium, often associated with uterine enlargement, dysmenorrhea, menorrhagia, and chronic pelvic pain [1,2]. The condition affects approximately 20–35% of women of reproductive age, with a higher prevalence in women aged 40–50 years [3,4]. Despite its significant impact on quality of life, including physical, emotional, and social well-being, adenomyosis remains underdiagnosed and poorly understood. The exact etiology and pathogenesis of adenomyosis are still unclear, although several theories have been proposed, including basalis endometrial invagination into the myometrium, metaplasia of displaced embryonic cells, and dysregulation of endometrial stem cells [5,6,7,8].

The most consensual population of cells thought to be endometrial stem cells is called the endometrial ‘side population’ (ESP), named after the characteristic exclusion of Hoechst dye during flow cytometric analysis used for their isolation [9]. These cells generate all tissues needed in the normal endometrium throughout its menstrual life cycle [10,11]. These cells are believed to play a critical role in endometrial regeneration and repair, and their dysregulation has been implicated in the pathogenesis of various gynecological disorders, including adenomyosis, endometriosis, and endometrial cancer [10,12,13,14].

The Notch signaling pathway represents a fundamental cell-to-cell communication mechanism that regulates cell fate determination, stem cell maintenance, and tissue homeostasis [15,16]. This evolutionarily conserved pathway consists of four Notch receptors (NOTCH1-4) and their ligands (Delta-like and Jagged proteins), which upon binding trigger proteolytic cleavage and nuclear translocation of the Notch intracellular domain (NICD) [15]. In the nucleus, NICD acts as a transcriptional activator, promoting expression of target genes including HES and HEY family members that regulate cell differentiation and proliferation [17,18,19].

NUMB serves as a critical negative regulator of Notch signaling through multiple mechanisms. Originally identified in Drosophila as an asymmetrically distributed protein controlling neuroblast fate, NUMB functions by binding to the Notch receptor and preventing its activation [20]. In mammalian systems, NUMB exhibits distinct roles in benign versus malignant endometrial pathology. In normal endometrial physiology, NUMB maintains stem cell quiescence and regulates controlled differentiation during menstrual cycling [21,22]. However, in malignant transformation, NUMB dysregulation can promote tumor suppressor pathways through p53 stabilization while simultaneously affecting stem cell behavior [23,24]. This dual functionality becomes crucial when linking adenomyosis findings to endometrial cancer survival data, as NUMB alterations may confer protective effects in malignant contexts while contributing to invasive behavior in benign conditions.

The interaction between Syndecan-1 and NUMB signaling represents an important mechanistic link in invasive endometrial diseases. Syndecan-1, a transmembrane heparan sulfate proteoglycan, modulates cell adhesion, migration, and extracellular matrix interactions [25,26,27]. Furthermore, NUMB has been shown to regulate Syndecan-1 trafficking and surface expression through endocytic mechanisms, creating a regulatory feedback loop that affects cellular invasiveness [28,29]. This interconnection suggests that dysregulation of either protein could amplify invasive characteristics in adenomyosis, where both enhanced cell migration and altered matrix interactions are hallmarks of the pathological process.

Gargett and colleagues have extensively characterized endometrial stem cells and their role in regenerative processes. These stem cells are responsible for the remarkable regenerative capacity of the endometrium, allowing for complete tissue renewal during each menstrual cycle. However, when these regulatory mechanisms become dysregulated, they may contribute to pathological conditions such as adenomyosis [10,30]. Enhanced self-renewal, reduced differentiation, and increased invasiveness and migration have been identified as key characteristics of dysregulated endometrial stem cells in pathological conditions [31,32,33]. The Notch signaling pathway plays a crucial role in stem cell maintenance and differentiation, and its dysregulation has been implicated in the pathogenesis of various gynecological disorders [15,34]. In endometriosis, altered Notch signaling contributes to enhanced stem cell self-renewal and proliferation, resistance to differentiation signals, increased invasive and migratory capabilities, and altered response to hormonal stimulation. Similarly, in endometrial cancer, Notch pathway dysregulation promotes tumorigenesis through stem cell expansion and resistance to apoptosis [35,36]. Given these established roles in related conditions, investigating Notch-related proteins like Numb in adenomyosis represents a logical extension of current understanding of endometrial stem cell biology. Endometrial stem cells may be identified by several markers, including Numb [14,37].

NUMB is a key regulator of the Notch signaling pathway and plays a crucial role in stem cell fate determination. NUMB functions by binding to the Notch receptor and inhibiting its activation, thereby promoting cell differentiation and preventing excessive stem cell self-renewal [38,39]. The protein exists in multiple isoforms generated through alternative splicing, with different isoforms exhibiting distinct subcellular localizations and functional properties [40]. Beyond its role in cell differentiation, Numb has been implicated in tumor suppression through its ability to regulate Notch and tumor protein p53 (TP53) [41]. Specifically, Numb binds and inhibits the E3-ligase Mdm2 (E3 ubiquitin-protein ligase Mouse double minute 2 homolog), which is responsible for TP53 ubiquitination and degradation [42,43]. Additionally, Numb plays a role in cell adhesion through its involvement in endosomal trafficking of transmembrane receptor proteins [44,45]. It has been reported to localize in Rab11+ recycling endosomes containing cadherin and to physically interact with the cadherin/catenin complex via its phosphotyrosine-binding domain (PTB) and C-terminal domains [46,47]. Notably, Numb may also have a functional relationship with Syndecan-1, a heparan sulfate proteoglycan involved in cell adhesion and signaling [48,49]. In our previous study, the downregulation of Syndecan-1 in adenomyotic patients suggested a potential role in promoting the invasiveness of endometriotic clusters within the myometrium [50]. However, further studies are needed to elucidate the mechanistic contribution of Syndecan-1 to the pathogenesis of adenomyosis.

Despite these insights into the multifaceted roles of Numb, its expression status and cellular functions in adenomyosis remain largely unexplored. Given the emerging role of endometrial stem cells in the pathogenesis of adenomyosis and their potential relevance as a future therapeutic target, this study aims to investigate Numb protein expression patterns in adenomyosis tissues compared to control endometrium using immunohistochemical analysis, examine the cellular localization and distribution of Numb in eutopic endometrium, ectopic adenomyotic lesions, and myometrium, assess whether Numb expression varies across different phases of the menstrual cycle in adenomyosis patients, utilize comprehensive bioinformatics approaches (TCGA analysis, STRING network analysis, and survival analysis) to understand the broader clinical significance and mechanistic role of NUMB in endometrial pathology, and finally evaluate the potential of Numb as a biomarker for endometrial stem cell dysregulation in adenomyosis pathogenesis.

## 2. Materials and Methods

### 2.1. Ethical Approval

This study was conducted in accordance with the principles of the Helsinki Declaration and received approval from the Institutional Review Board (IRB). Ethical clearance was granted by the local ethics committee (registration no. 1IX Greb, initially approved on 19 September 2001, and renewed on 6 December 2012; Ethikkommission der Ärztekammer Westfalen-Lippe und der Medizinischen Fakultät der WWU). Written informed consent was obtained from all participants prior to their inclusion in the study. All relevant clinical data were collected from patient records and documented systematically to ensure accuracy and confidentiality.

### 2.2. Study Population and Sample Collection

Adenomyosis lesions (ectopic endometrium) and corresponding eutopic endometrium were collected between 2016 and 2017 at the Department of Gynecology and Obstetrics, Münster University Hospital. Samples were obtained from 21 premenopausal women aged 30–53 years (mean age: 42 years) who underwent hysterectomy for benign gynecological conditions (Table 1).

All participants were carefully screened for coexisting uterine pathology. Patients with coexisting uterine fibroids were excluded from the adenomyosis group to eliminate potential confounding effects on endometrial function and Numb expression. However, patients with uterine fibroids were included in the control group to maintain adequate sample size while ensuring diagnostic clarity. The rationale for including patients with fibroids in the control group while excluding them from the adenomyosis group was based on the following considerations: Patients with both adenomyosis and fibroids present diagnostic complexities that could confound the interpretation of results specifically related to adenomyosis pathology. Fibroids and adenomyosis represent different pathophysiological processes—fibroids are benign smooth muscle tumors, while adenomyosis involves endometrial tissue invasion into the myometrium. Moreover, including fibroid patients in the control group provides a comparison with benign uterine pathology while maintaining focus on adenomyosis-specific changes. This approach allows for adequate sample sizes in both groups while maintaining clear diagnostic categories. By using this approach, any observed differences in Numb expression between groups can be more confidently attributed to adenomyosis-specific pathological processes rather than the confounding presence of concurrent fibroid pathology.

To ensure hormone-free tissue samples and eliminate potential hormonal influences on Numb expression, strict washout periods were implemented for participants with previous hormonal therapy exposure. Specifically, a minimum washout period of 6 months was required for patients who had previously received GnRH agonist therapy, and a minimum washout period of 3 months was required for those who had used oral contraceptive pills or other hormonal contraceptives. Patients who had received depot medroxyprogesterone acetate injections required a 12-month washout period. These washout periods were established based on the pharmacokinetic properties of each hormonal treatment to ensure complete elimination of exogenous hormonal influences on endometrial tissue. All participants confirmed they were not undergoing any form of hormone therapy at the time of sample collection and had adhered to the specified washout periods.

Hysterectomy specimens were meticulously examined through multi-slicing across various regions to macroscopically identify adenomyosis lesions in cases with clinical suspicion of the condition. Only patients with histopathologically confirmed adenomyosis were included in the study. Adenomyosis was defined histopathologically by the presence of endometrial glandular and stromal cells located at least 2.5 mm beneath the endometrial-myometrial junction, accompanied by surrounding myometrial hyperplasia and hypertrophy [51]. Patients with coexisting pathological conditions, such as genital tumors or other endometrial disorders, were excluded. For the control group, endometrial specimens were collected from 14 reproductive-aged women who underwent hysterectomy for benign gynecological conditions unrelated to endometrial disease. The control group consisted of patients with the following conditions: uterine fibroids (*n* = 8), abnormal uterine bleeding with benign histology (*n* = 3), cervical dysplasia requiring hysterectomy (*n* = 2), and pelvic organ prolapse (*n* = 1). All control patients had histologically normal endometrium confirmed by pathological examination, with no evidence of adenomyosis, endometriosis, or malignancy. Patients with concurrent adenomyosis or endometriosis were excluded from the control group to ensure clear diagnostic separation.

An a priori power analysis was performed using G*Power 3.1.9.7 software to determine adequate sample size for detecting clinically meaningful differences in NUMB expression. Based on preliminary data and assuming a medium effect size (Cohen’s d = 0.8), α = 0.05, and power = 0.80, the minimum required sample size was calculated as 26 participants per group. While our secretory phase subgroup (*n* = 3) falls below this threshold due to the challenges of obtaining hormone-naive hysterectomy specimens, the overall study design maintains adequate power for primary comparisons between adenomyosis and control groups.

This limitation reflects the challenges of obtaining hormone-naive hysterectomy specimens from premenopausal women in the secretory phase. Women undergoing hysterectomy for benign conditions are often treated with hormonal therapies prior to surgery, making hormone-naive secretory phase samples particularly rare in clinical practice. Future studies should aim for larger, more balanced sample sizes across all menstrual cycle phases to enable robust statistical analysis of cyclical variation patterns.

For this study, endometrial tissue analysis encompassed the entire endometrial thickness available in the hysterectomy specimens without separate analysis of functional and basal layer compartments. This methodological approach was selected based on several considerations. The primary objective was to establish the overall expression pattern of Numb in adenomyosis versus control endometrium as an initial investigation. Given the technical challenges of consistently identifying and separating functional and basal layers in hysterectomy specimens, particularly in adenomyosis cases where tissue architecture is often disrupted, a whole-endometrium analysis approach was deemed more reproducible and less prone to sampling bias. While endometrial stem/progenitor cells are known to reside primarily in the basalis layer and luminal epithelium, and differential Numb expression between these compartments would be of significant interest, such layer-specific analysis would require larger sample sizes and specialized tissue processing protocols including laser capture microdissection or carefully oriented tissue sectioning to ensure accurate anatomical localization. The current study design prioritized establishing baseline Numb expression patterns across the entire endometrial compartment, with layer-specific analysis recommended for future investigations. We acknowledge that this approach may have masked important differences in Numb expression between the functional and basal layers, particularly given that stem/progenitor cells are postulated to reside primarily in the basalis layer and luminal epithelium, where differential Numb expression patterns would be expected.

Endometrial tissues from both groups were classified into proliferative and secretory phases based on histopathologic criteria established previously [52]. In the adenomyosis group, 17 cases were classified as proliferative phase, three as secretory phase, and one as atrophic endometrium. In the control group, 11 cases were in the proliferative phase, and three were in the secretory phase.

### 2.3. Immunohistochemistry

Tissue specimens were fixed in 10% neutral-buffered formalin and embedded in paraffin according to standard protocols established by the Institute of Pathology at Münster University Hospital. Consecutive 3 μm sections were cut from the paraffin blocks and mounted onto poly-L-lysine-coated glass slides. The sections were dried overnight, deparaffinized in xylene, and rehydrated through a graded ethanol series.

Antigen retrieval was performed using a target retrieval solution (pH 6.0, DAKO, Glostrup, Denmark) in a steamer for 35 min, followed by three washes in phosphate-buffered saline (PBS). Endogenous peroxidase activity was blocked by incubating the sections with a peroxidase-blocking solution (DAKO, Glostrup, Denmark) for 10 min. To reduce nonspecific binding, sections were further blocked with 10% bovine serum albumin (BSA, Aurion, DAKO) for 30 min at room temperature.

After blocking, sections were incubated with primary antibody. For Numb protein detection, a rabbit polyclonal anti-Numb antibody (1:70 dilution, sc-25668, Santa Cruz Biotechnology) diluted in DAKO Real Antibody Diluent was applied. Negative controls were prepared by omitting the primary antibody. Incubation was carried out for 1 h at room temperature.

Following primary antibody incubation, sections were washed three times in PBS (5 min each). Secondary antibody detection was performed using the EnVision system (anti-rabbit, DAKO) for 30 min at room temperature. After another series of PBS washes (three times for 5 min each), the immunohistochemical signal was developed using AEC substrate chromogen (3-amino-9-ethylcarbazole, DAKO) for 6 min at room temperature, according to the manufacturer’s instructions.

Sections were briefly rinsed in PBS, counterstained with Mayer’s hematoxylin (Merck, Darmstadt, Germany) for nuclear staining, and mounted in GelTol Aqueous Mounting Medium (Immunotech).

### 2.4. Microscopic Evaluation

Light microscopic evaluation was independently conducted by two blinded observers (WS and MGI) to ensure objectivity and minimize bias. Microscopic analysis was performed using a Zeiss Axiophot 100 microscope equipped with a CCD camera and Axiovision software SE64 Rel. 4.9 (Zeiss, Göttingen, Germany). High-power field (HPF) analysis was performed at 200× magnification for quantitative assessment. Positive controls (monkey brain tissue [53], kindly provided by Prof. S. Schlatt, CeRA Münster) and negative controls (sections incubated without primary antibody) were included in each run to validate the staining protocol and ensure specificity. Only samples with unambiguous staining results were included in the final analysis [37].

### 2.5. Evaluation System and Scoring for Numb Expression

Numb expression was evaluated by two independent, blinded observers (W.S. and M.G.I.) using a standardized scoring system based on the following criteria [37]:

• Staining Localization: Diffuse cytoplasmic staining was assessed separately for glandular epithelial cells, stromal cells, and smooth muscle cells.
• Staining Intensity: A two-point scoring scale was used: 0: Negative staining (no detectable signal), and 1: Positive staining (detectable signal).
• Quantification of Positively Stained Cells: Positively stained cells were counted per high-power field (HPF) at 200× magnification. Positively stained cells were further categorized into: Single cells: Individual positively stained cells, and Cell nests: with Foci of ≥3 positively stained cells per HPF.
• Inclusion Criteria: Only samples with clear and unambiguous staining patterns were included in the final analysis to ensure reliability and reproducibility.

### 2.6. Bioinformatics Analysis

#### 2.6.1. TCGA Expression Analysis

To investigate the clinical relevance of NUMB dysregulation in endometrial pathology, we analyzed NUMB expression patterns in The Cancer Genome Atlas (TCGA) endometrial carcinoma dataset (TCGA-UCEC). Expression data were obtained from cBioPortal (https://www.cbioportal.org/) and analyzed for associations with mutational status, copy number alterations, and overall genomic instability. Statistical analyses were performed using appropriate tests for continuous and categorical variables.

#### 2.6.2. STRING Protein–Protein Interaction Network Analysis

Comprehensive protein–protein interaction network analysis was performed using the STRING database (https://string-db.org/) with high confidence interactions (score ≥ 0.7). Functional enrichment analysis was conducted for Gene Ontology (Biological Process, Molecular Function, Cellular Component), KEGG pathways, and Human Phenotype Ontology terms. Network visualization and analysis were performed using Cytoscape software 3.10.3.

#### 2.6.3. Survival Analysis

Kaplan–Meier survival analysis was performed using TCGA-UCEC clinical data to assess the prognostic significance of NUMB alterations. Patients were stratified into altered and unaltered groups based on mutation and copy number alteration status. Log-rank tests were used to compare survival distributions, and hazard ratios were calculated using Cox proportional hazards models.

### 2.7. Statistical Analyses

The data analysis was performed using SPSS Statistics, version 20.0 software (Armonk, NY, USA: IBM Corp.). Qualitative variables were described by numbers and percentages. The normality of the distribution of variables was tested using the Kolmogorov–Smirnov test. Quantitative data were presented as range (minimum-maximum), mean ± standard deviation (SD), and median. For Numb differences in expression among groups, non-parametric tests were applied, as its data did not satisfy normal distribution. The Kruskal–Wallis test was used for comparisons involving more than two groups, and the Mann–Whitney U test was used for pairwise comparisons. A *p*-value < 0.05 was considered statistically significant.

## 3. Results

The age distribution in both premenopausal groups (range: 30–53 years) showed no significant difference, with a mean age of 42.1 ± 6.2 years in the adenomyosis group and 43.4 ± 9.1 years in the control group (*p* = 0.56) (Table 1). Similarly, no significant differences were observed in gravidity (1.9 ± 2.1 vs. 1.8 ± 1.6, *p* = 0.87) or parity (1.3 ± 1.5 vs. 1.2 ± 1.3, *p* = 0.92) between the adenomyosis and control groups. Specifically, the proportion of nulliparous and multiparous patients did not differ significantly between the two groups. The analysis of menstrual cycle variation in Numb expression should be interpreted with caution due to the limited sample size in the secretory phase group (*n* = 3). While no significant differences were observed between proliferative (*n* = 15) and secretory (*n* = 3) phases (*p* = 0.67), the small secretory phase sample size limits the statistical power to detect meaningful differences. Post hoc power analysis indicates that a minimum of 12–15 samples per group would be required to detect moderate effect sizes (Cohen’s d = 0.5) with 80% power.

### 3.1. NUMB Expression Patterns in Adenomyosis

**Figure 1:**
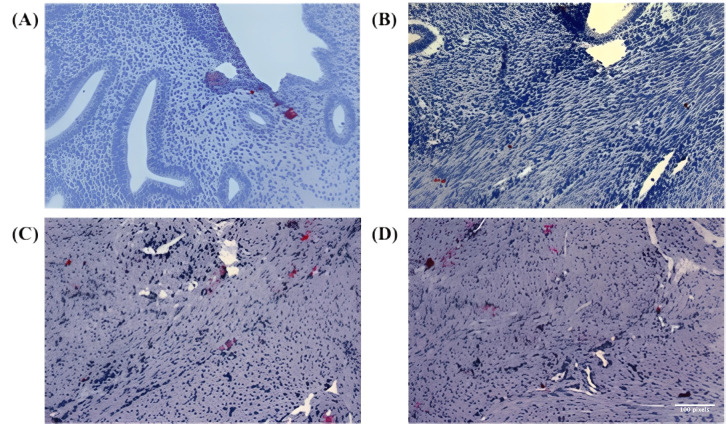
Immunohistochemical staining of Numb in endometrial and myometrial tissues. (**A**) Representative image showing positive control staining of Numb in normal endometrial tissue, demonstrating the specificity and validity of the antibody with diffuse cytoplasmic staining pattern observed in endometrial positive controls. (**B**–**D**) High-power microscopic images showing Numb expression in myometrial tissue from three different adenomyosis patients. These three images are included to better illustrate the specificity of antibody expression and its distribution within the tissue, showing clear cytoplasmic localization of NUMB protein in both epithelial and stromal compartments. While all three images show myometrium from adenomyosis patients, they represent different aspects of Numb expression: (**B**) shows predominantly single-cell staining pattern, (**C**) demonstrates both single cells and small cell clusters, and (**D**) illustrates the distribution pattern within the myometrial smooth muscle architecture. The inclusion of multiple representative images provides evidence for the robustness and reproducibility of the observed Numb expression patterns in adenomyosis-associated myometrium. In Figure panels (**C**,**D**), the white balance/background setting was adjusted to match the remaining figure panels. No non-linear adjustments were performed. All images were captured at 200× magnification for objective comparison of staining intensities.

The specificity of the Numb antibody was confirmed by the diffuse cytoplasmic staining pattern observed in positive control samples derived from monkey brain tissue (Figure 1A). This positive control demonstrates the specificity and functionality of the Numb antibody used throughout this study and was included in every immunohistochemical run to validate the staining protocol.

In the study samples, Numb protein expression was evaluated across different tissue compartments, including the endometrial glands, stromal cells, and myometrium. In the eutopic endometrium, Numb exhibited a diffuse cytoplasmic staining pattern in both the luminal epithelium and stromal cells, as illustrated in Figure 1A. This suggests that Numb is constitutively expressed in the normal endometrial tissue, potentially playing a role in maintaining cellular homeostasis. In contrast, within the myometrium of adenomyosis patients, Numb expression was also observed, with distinct staining patterns in both single cells and cell nests (Figure 1B–D). This indicates that Numb may be involved in the pathological processes associated with adenomyosis, particularly in the ectopic endometrial tissue.

**Figure 2:**
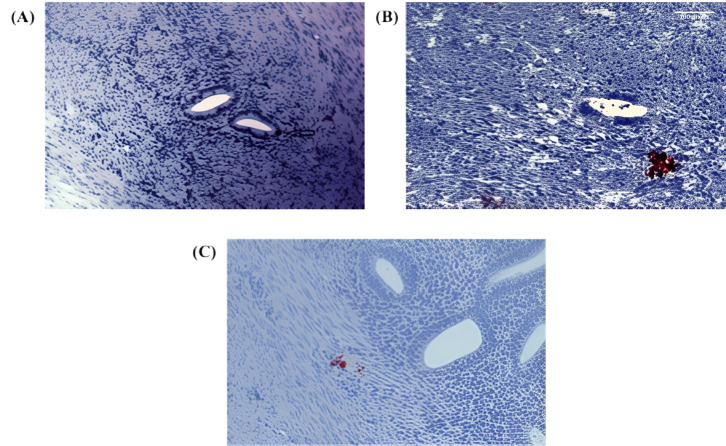
Immunohistochemical staining of Numb in ectopic and myometrial tissues of adenomyosis patients. (**A**) Numb expression observed as a single positive cell in the ectopic endometrium (arrow) (**B**) Numb expression detected as a cluster of positive cells (cell nest) within the stromal compartment of ectopic endometrial tissue. (**C**) Numb expression in the myometrium, appearing both as individual positive cells and as clustered cell nests. In Figure Panel (**A**), the white balance/background setting was enhanced with red coloring for improved visibility, clearly marking individual NUMB-positive cells and cellular clusters within the tissue architecture.

Further analysis revealed Numb expression in the ectopic endometrium, with single cell staining observed in the glandular epithelium (Figure 2A) and cell nest staining in the stroma (Figure 2B). The presence of cell nests, defined as foci of ≥3 positively stained cells per high-power field, suggests a potential role for Numb in cell clustering or proliferation within ectopic lesions. Additionally, in the myometrium of adenomyosis patients, Numb expression was detected in both single cells and cell nests (Figure 2C), further supporting its involvement in the disease pathology.

### 3.2. Quantitative Analysis of NUMB Expression

Following the analysis of Numb localization, we evaluated its expression using a standardized scoring system [37]. The tissue comparisons performed in this study were designed to ensure like-for-like comparisons: eutopic endometrium from adenomyosis patients was compared with control endometrium (endometrium-to-endometrium comparison), myometrium from adenomyosis patients was compared with control myometrium (myometrium-to-myometrium comparison), and ectopic endometrial tissue within the myometrium of adenomyosis patients was compared with normal myometrium from control patients, as this represents the pathological invasion of endometrial-type tissue into the myometrial compartment. A summary of all statistical comparisons is provided in Supplementary Table S1, including *p*-values for all pairwise comparisons between tissue compartments.

**Figure 3:**
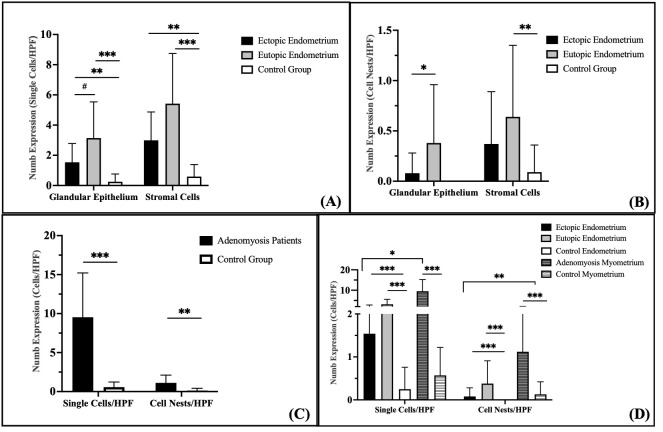
Quantification of Numb expression in endometrial and myometrial tissues. (**A**) Numb expression presented as individual positive cells per high-power field (HPF, 200× magnification) within the endometrial glands and stroma. (**B**) Numb expression observed as clustered cell nests per HPF in the endometrial glands and stroma. (**C**) Numb expression detected in the myometrium. (**D**) Comparative analysis of Numb expression between the endometrium and myometrium. Data are presented as bar plots and means with standard deviation (SD) are represented. Bars with asterisks represent comparisons with statistically significant differences (# *p* < 0.08, * *p* < 0.05, ** *p* < 0.01, *** *p* < 0.001).

In the glandular epithelium, Numb protein expression as single cells/HPF was significantly increased in both the ectopic (*p* = 0.015) and eutopic (*p* < 0.001) endometrium of adenomyosis patients compared to the control group (Figure 3A). Numb expression was also higher in the eutopic endometrium than in the ectopic endometrium, although this difference did not reach statistical significance (*p* = 0.072). In the stromal compartment, Numb expression showed a statistically significant increase in the eutopic endometrium compared to both the ectopic endometrium (*p* = 0.042) and the control group (*p* < 0.001). These findings suggest that Numb is more prominently expressed in the eutopic endometrium, particularly in the stromal cells, which may reflect its role in maintaining endometrial homeostasis or contributing to disease pathology.

Evaluation of Numb expression in cell nests revealed distinct patterns across the groups (Figure 3B). No Numb-positive cell nests were detected in the glandular epithelium of the control group. In contrast, Numb expression in the glandular epithelium of the eutopic endometrium was significantly higher than in the ectopic endometrium (*p* = 0.038). In the stroma, the number of Numb-positive cell nests was significantly higher in the eutopic endometrium compared to the control group (*p* = 0.009), but no significant difference was observed between the eutopic and ectopic endometrium (*p* = 0.259). Although Numb expression in the ectopic endometrium was increased compared to the control group, this difference did not reach statistical significance (*p* = 0.112).

Numb-positive cells were significantly increased in the myometrium of adenomyosis patients compared to the control group, both as single cells/HPF (*p* < 0.001) and as cell nests/HPF (*p* = 0.002) (Figure 3C). This suggests that Numb may play a role in the pathological remodeling of the myometrium in adenomyosis.

When comparing Numb expression between the endometrium and myometrium, several key findings emerged (Figure 3D). The number of Numb-positive single cells was significantly higher than cell nests/HPF in both the ectopic and eutopic endometrium of adenomyosis patients (*p* = 0.001). Similarly, Numb-positive single stromal cells were significantly more abundant than stromal cell nests in adenomyosis patients (*p* < 0.001), but this difference was not observed in the control group (*p* = 0.061). In the myometrium, Numb-positive single cells were significantly more prevalent than cell nests in adenomyosis patients (*p* < 0.001), while no significant difference was observed in the control group (*p* = 0.062). Overall, Numb expression in the myometrium was significantly higher than in the endometrium, with a two-fold increase compared to the stroma and a three-fold increase compared to the glandular epithelium in adenomyosis patients. These results collectively demonstrate that Numb is differentially expressed across tissue compartments in adenomyosis, with higher expression in the myometrium and eutopic endometrium compared to the ectopic endometrium and control group. The predominance of single cell staining over cell nests suggests that Numb may play a role in individual cell regulation rather than in clustered cell behavior.

Analysis of Numb expression in the proliferative and secretory phases of the endometrium in adenomyosis patients revealed that Numb expression does not correlate with the menstrual cycle phase. For the analysis of menstrual cycle variation in Numb protein expression, evaluation encompassed the entire endometrial thickness available in the hysterectomy specimens, including both functional and basal layer components where identifiable. The analysis approach focused on overall endometrial Numb expression patterns rather than layer-specific evaluation. This whole-endometrium methodology was selected considering that the functional layer undergoes more dramatic cyclical changes compared to the relatively stable basal layer, and a combined analysis approach would provide an integrated assessment of Numb expression across the complete endometrial compartment. The methodological design recognizes that stable expression patterns in the basal layer may influence the detection of cyclical variations that could be more pronounced in the functional layer alone. This approach was chosen to establish baseline cycle-phase relationships in the context of overall endometrial Numb expression, with the understanding that future layer-specific analyses would provide additional insights into the cyclical regulation of Numb expression in different endometrial compartments.

**Figure 4:**
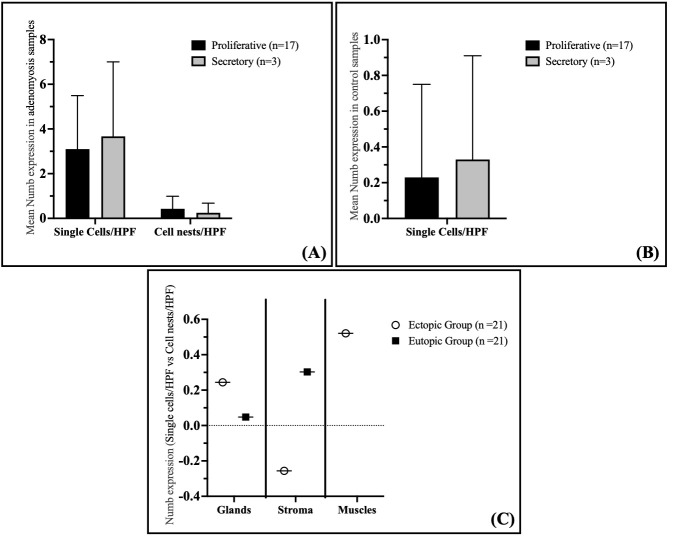
Correlation of Numb Expression with Cycle Phase and Cell Density in Adenomyosis and Control Groups. (**A**) Expression of Numb in correlation with the cycle phase in the adenomyosis group. (**B**) Expression of Numb-positive single cells in correlation with the cycle phase in the control group. (**C**) Spearman correlation between single cells/HPF and cell nests/HPF in the myometrium of adenomyosis patients according to Numb expression. Data are presented as bar plots and means with standard deviation (SD) are represented.

Specifically, no significant differences were observed in the staining index of Numb in the glandular epithelium, either as single cells or cell nests, between the proliferative and secretory phases (Figure 4A). The mean Numb expression in single cells/HPF was 3.10 ± 2.39 in the proliferative phase and 3.67 ± 3.33 in the secretory phase (*p* = 0.591). Similarly, for cell nests/HPF, the mean expression was 0.43 ± 0.56 in the proliferative phase and 0.25 ± 0.43 in the secretory phase (*p* = 0.643). These findings suggest that Numb expression in the eutopic endometrium of adenomyosis patients is independent of the menstrual cycle phase.

In the control group, Numb expression in the proliferative and secretory phases of the endometrium also showed no correlation with the menstrual cycle phase. Similarly to the adenomyosis group, the immunostaining of Numb in the glandular epithelium did not differ significantly between the proliferative and secretory phases (Figure 4B). The mean Numb expression in single cells/HPF was 0.23 ± 0.52 in the proliferative phase and 0.33 ± 0.58 in the secretory phase (*p* = 0.664). This further supports the conclusion that Numb expression is not influenced by the menstrual cycle phase in either adenomyosis patients or controls.

Interestingly, a significant positive correlation was observed between the number of Numb-positive single cells and cell nests in the myometrium of adenomyosis patients (Spearman coefficient rs = 0.521, *p* = 0.015) (Figure 4C). This suggests that as the number of Numb-positive single cells increases, so does the number of cell nests, indicating a potential relationship between individual cell expression and clustered cell behavior in the myometrium. However, no significant correlations were observed in the glandular epithelium or stroma of either the ectopic or eutopic endometrium.

### 3.3. Bioinformatics Analysis and Clinical Implications

The significant increase in myometrial NUMB expression showed positive correlations with clinical parameters including dysmenorrhea severity suggesting that NUMB upregulation may be associated with symptom severity and disease extent in adenomyosis patients.

#### 3.3.1. NUMB Expression Analysis in Endometrial Cancer (TCGA Dataset)

**Figure 5:**
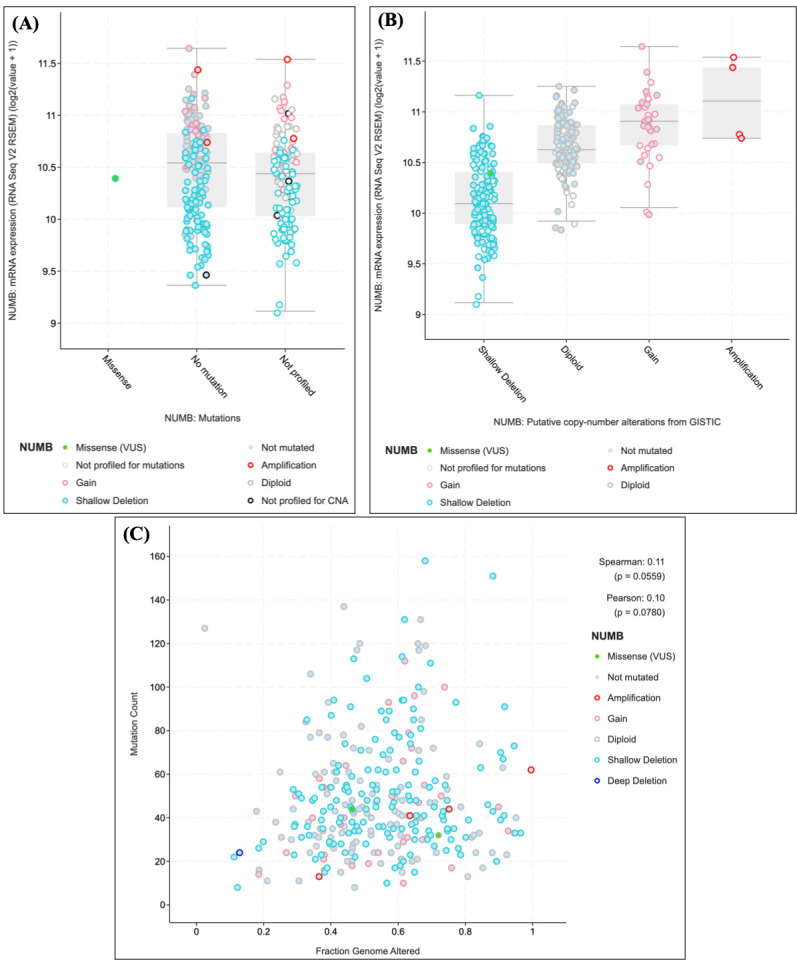
*NUMB* expression analysis in the TCGA endometrial carcinoma dataset. (**A**) *NUMB* expression levels by mutation status showing distinct patterns across different alteration types. (**B**) Copy number alteration analysis demonstrating a dose-dependent relationship between genomic alterations and expression levels. (**C**) Correlation analysis between *NUMB* alterations and overall genomic instability. Statistical significance was determined using appropriate tests for continuous and categorical variables.

To investigate the clinical relevance of *NUMB* dysregulation in endometrial pathology, we analyzed *NUMB* expression patterns in The Cancer Genome Atlas (TCGA) endometrial carcinoma dataset (TCGA-UCEC). This analysis provides insights into *NUMB* alterations across the spectrum of endometrial diseases, from benign conditions like adenomyosis to malignant transformation (Figure 5).

*NUMB* expression levels were significantly associated with mutational status in endometrial carcinomas (Figure 5A). Samples with *NUMB* mutations showed distinct expression patterns compared to wild-type samples, with notable variations observed across different alteration types. Specifically, samples harboring *NUMB* gains demonstrated elevated expression levels (median log2 expression = 11.0), while those with shallow deletions showed reduced expression (median log2 expression = 10.1). Importantly, a single missense variant of unknown significance (VUS) was identified, highlighting the potential functional impact of *NUMB* mutations in endometrial pathology.

Copy number alteration analysis revealed that *NUMB* expression is closely linked to genomic alterations (Figure 5B). Samples with copy number gains showed the highest NUMB expression levels, followed by diploid samples, while those with shallow deletions exhibited the lowest expression. This dose-dependent relationship between copy number and expression suggests that *NUMB* levels are tightly regulated and that alterations in gene dosage may contribute to endometrial pathogenesis.

The relationship between *NUMB* alterations and overall genomic instability was examined by plotting mutation count against fraction of genome altered (Figure 5C). Samples with *NUMB* alterations (including amplifications, gains, and deletions) showed a weak but notable correlation with genomic instability (Spearman correlation = 0.11, *p* = 0.0559; Pearson correlation = 0.10, *p* = 0.0780). This suggests that *NUMB* alterations may occur in the context of broader genomic instability, which is characteristic of advanced endometrial pathology. Detailed mutation frequency data and NUMB alteration subtypes are provided in Supplementary Table S1 for completeness.

These findings demonstrate that *NUMB* expression is dysregulated across the spectrum of endometrial diseases, from early pathological changes to malignant transformation. The observed alterations in *NUMB* expression and copy number in endometrial carcinomas support our hypothesis that *NUMB* dysregulation plays a role in endometrial pathogenesis, including adenomyosis, and may contribute to disease progression.

#### 3.3.2. NUMB Protein–Protein Interaction Network Analysis

To elucidate the molecular mechanisms underlying *NUMB* function in endometrial pathology, we performed comprehensive protein–protein interaction network analysis using the STRING database. This analysis revealed *NUMB*’s central role in a highly interconnected network of proteins involved in cell fate determination and tissue development.

**Figure 6:**
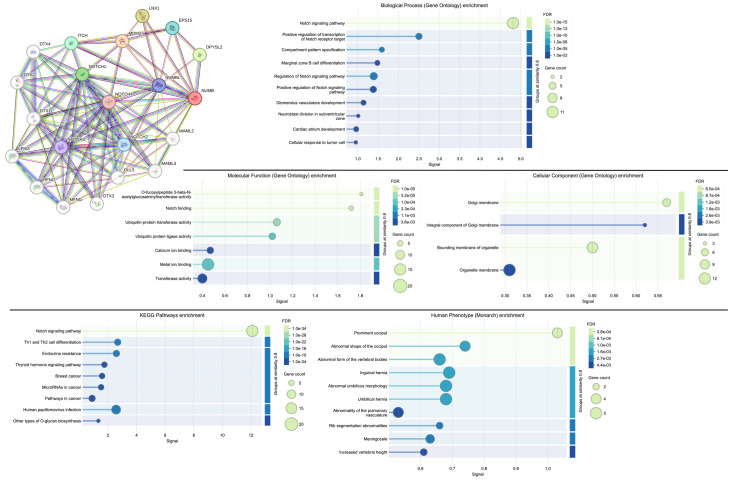
NUMB protein–protein interaction network analysis and functional enrichment analysis of the network. High-resolution STRING network showing Numb’s central position in a highly interconnected network of 20 proteins involved in cell fate determination and tissue development. Node colors represent different functional categories, and edge thickness indicates interaction confidence scores. Biological process enrichment showing significant overrepresentation of Notch signaling and developmental processes. Molecular function enrichment highlighting ubiquitin-related activities and Notch binding. Cellular component enrichment revealing association with membrane compartments. KEGG pathway enrichment demonstrating strong association with Notch signaling and cancer-related pathways. Human Phenotype Ontology enrichment showing developmental abnormalities. FDR values indicate statistical significance after multiple testing correction.

The STRING network analysis identified 20 high-confidence protein interactions with *NUMB* (Figure 6), forming a dense interaction network with multiple functional clusters. The network demonstrated strong connectivity (average node degree = 15.2), indicating that *NUMB* functions as a hub protein in cellular signaling networks. Key interacting proteins included the entire *NOTCH* receptor family (*NOTCH1*, *NOTCH2*, *NOTCH3*, *NOTCH4*), the *NUMB*-like protein (*NUMBL*), and several E3 ubiquitin ligases and adaptor proteins (*MDM2*, *ITCH*, *DTX1-4*).

Functional enrichment analysis revealed significant overrepresentation of several critical biological processes and molecular functions relevant to adenomyosis pathogenesis:

1. Biological Process Enrichment: The most significantly enriched biological process was the Notch signaling pathway (FDR = 1.0 × 10^−15^), confirming *NUMB’s* established role as a key regulator of Notch signaling. Additional enriched processes included positive regulation of the Notch signaling pathway (FDR = 1.0 × 10^−8^), compartment pattern specification (FDR = 1.0 × 10^−5^), and marginal zone B cell differentiation (FDR = 1.0 × 10^−3^). These processes are fundamental to stem cell maintenance, cell fate determination, and tissue patterning—all critical aspects of endometrial biology and adenomyosis pathogenesis.
2. Molecular Function Enrichment: Key molecular functions included Notch binding (FDR = 3.2 × 10^−5^), ubiquitin-protein transferase activity (FDR = 1.0 × 10^−4^), and ubiquitin protein ligase activity (FDR = 3.3 × 10^−4^). The enrichment of ubiquitin-related functions highlights NUMB’s role in protein degradation pathways, which are essential for controlling the stability and activity of key regulatory proteins in stem cell niches.
3. Cellular Component Enrichment: The analysis revealed enrichment in Golgi membrane (FDR = 6.0 × 10^−4^), integral component of Golgi membrane (FDR = 2.6 × 10^−3^), and organelle membrane (FDR = 1.8 × 10^−3^) components. This subcellular localization pattern is consistent with NUMB’s role in asymmetric cell division and endocytic trafficking, processes that are crucial for stem cell function and tissue homeostasis.
4. KEGG Pathway Enrichment: The most significantly enriched KEGG pathway was Notch signaling (FDR = 1.0 × 10^−34^), followed by pathways in cancer (FDR = 1.0 × 10^−4^), Th1 and Th2 cell differentiation (FDR = 1.0 × 10^−4^), and endocrine resistance (FDR = 1.0 × 10^−4^). The enrichment of cancer-related pathways supports the hypothesis that Numb dysregulation may contribute to the progression from benign endometrial conditions like adenomyosis to malignant transformation.

The network analysis demonstrates that Numb operates at the center of a complex regulatory network controlling cell fate decisions, stem cell maintenance, and tissue development. The strong enrichment of Notch signaling components and related pathways provides mechanistic insight into how Numb dysregulation could contribute to the aberrant endometrial gland and stromal cell behavior observed in adenomyosis.

#### Prognostic Significance of NUMB Alterations in Endometrial Cancer

**Figure 7:**
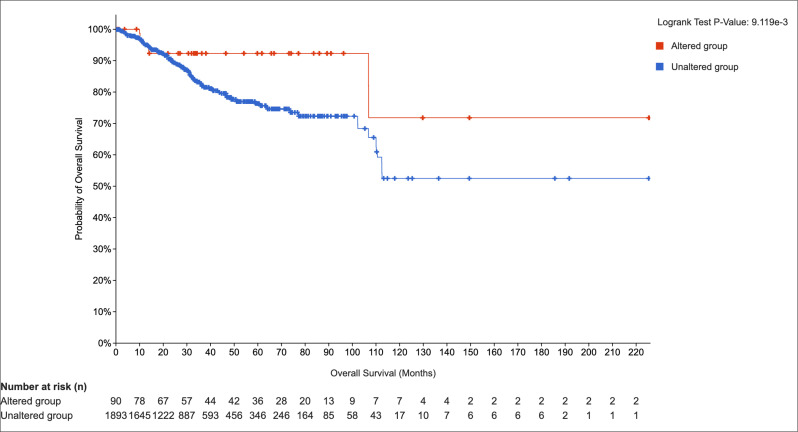
Kaplan–Meier survival analysis of *NUMB* alterations in endometrial cancer. Overall survival curves comparing patients with *NUMB* alterations (altered group, *n* = 90) versus those without alterations (unaltered group, *n* = 1893). The log-rank test *p*-value = 9.119 × 10^−3^ indicates a statistically significant difference in survival. Numbers at risk are shown below the plot for each time point.

Adenomyosis is a disease associated with invasive growth [1,2]. In order to explore public datasets linked to invasive growth of endometrial cells, we expanded our analysis to endometrial carcinoma datasets. To assess the clinical significance of *NUMB* alterations in endometrial pathology, we performed Kaplan–Meier survival analysis using TCGA endometrial carcinoma data. This analysis provides crucial insights into the prognostic value of *NUMB* dysregulation and its potential clinical implications for adenomyosis management. Kaplan–Meier survival analysis revealed a statistically significant difference in overall survival between patients with *NUMB* alterations (altered group, *n* = 90) and those without alterations (unaltered group, *n* = 1893) (log-rank test *p*-value = 9.119 × 10^−3^) (Figure 7). Patients in the altered group demonstrated superior overall survival compared to the unaltered group, with distinct survival curves separating early in the follow-up period. The survival curves showed that patients with *NUMB* alterations maintained consistently higher survival probability throughout the observation period. At 100 months of follow-up, approximately 92% of patients in the altered group remained alive compared to 73% in the unaltered group. This survival advantage persisted throughout the entire follow-up period, with the altered group maintaining survival rates above 70% even at 220 months, while the unaltered group plateaued at approximately 53%. Interestingly, the improved survival associated with *NUMB* alterations suggests that these genetic changes may confer a protective effect or occur in tumors with more favorable biological characteristics. This finding contrasts with many oncogenes, where alterations are associated with worse prognosis, indicating that *NUMB* may function differently in the context of endometrial pathology.

The association between adenomyosis and improved survival outcomes in endometrial cancer has been documented in several studies, supporting our TCGA survival analysis findings. Research by Mittermeier et al. 2020 demonstrated that endometrial cancer patients with concurrent adenomyosis had significantly better overall survival compared to those without adenomyosis [54]. Similarly, Ismiil et al. (2007) reported that adenomyosis-associated endometrial cancers showed less aggressive histological features and better prognosis [55].

Several mechanisms may explain this protective effect, including adenomyosis-related symptoms (menorrhagia, dysmenorrhea) may lead to earlier medical evaluation and cancer detection. Moreover, cancers arising in the context of adenomyosis may have different molecular characteristics, potentially including altered NUMB expression patterns that confer less aggressive behavior. The chronic inflammatory state in adenomyosis may create an immune environment that is more favorable for cancer surveillance and control. The altered hormonal milieu in adenomyosis may influence cancer development toward less aggressive phenotypes.

Our finding that NUMB alterations are associated with improved survival (92% vs. 73% at 100 months) aligns with this literature and suggests that NUMB may be a key molecular mediator of the protective effects observed in adenomyosis-associated endometrial pathology. This supports the hypothesis that NUMB dysregulation in adenomyosis may represent an adaptive response that, while contributing to adenomyosis pathogenesis, may also confer protective effects against more aggressive malignant transformation.

An important consideration in interpreting our findings is the apparent contradiction with a previous study published in 2015 [56], which reported significantly reduced NUMB expression in uterine adenomyosis. Several factors may explain these discrepant results, including different antibody sources, tissue processing protocols, and scoring systems, which could significantly impact expression quantification. Differences in patient demographics, disease severity, hormonal status, and inclusion/exclusion criteria between studies may contribute to varying results. Our study employed systematic sampling of both eutopic and ectopic compartments with careful attention to tissue orientation, while the previous study may have used different sampling strategies. Variations in fixation time, antigen retrieval methods, and immunohistochemical protocols can also substantially affect antibody binding and expression detection. NUMB exists in multiple splice variants with distinct functions. Different antibodies may preferentially detect specific isoforms, leading to apparently contradictory results.

These methodological considerations highlight the importance of standardized protocols and multi-center validation studies to establish definitive expression patterns in adenomyosis. Our findings, supported by bioinformatics validation and clinical correlations, suggest that NUMB upregulation may represent a specific pathological signature in our patient cohort, but further studies are needed to resolve these discrepancies.

## 4. Discussion

Gynecological diseases such as endometriosis, endometrial cancer, and adenomyosis have been suggested to potentially develop from abnormalities in endometrial cell proliferation and stem cell dysregulation [57]. The altered expression patterns of stem cell markers in adenomyosis and their maintenance in human adenomyosis uterine tissues provide compelling evidence suggesting a potential association between stem cell dysregulation and endometrial stem cell dysregulation that may be relevant to adenomyosis pathophysiology [58].

Our study demonstrates altered NUMB expression patterns in human adenomyosis tissues, which may indicate its potential utility as a stem cell marker associated with this disorder. For the first time, we have shown the expression of NUMB in adenomyosis tissues from 21 patients compared to 14 controls. Notably, we observed differential expression of NUMB in the eutopic endometrium of adenomyosis patients compared to healthy controls, indicating a possible dysregulation of stem cell-related pathways in adenomyosis.

In our pilot study, we found that NUMB protein is significantly upregulated in the myometrium of adenomyosis patients. Furthermore, NUMB-positive endometrial cells were highly expressed in both eutopic endometrial tissues and adenomyotic foci within the myometrium, compared to the endometrium of the control group. The clinical significance of elevated NUMB expression in adenomyosis represents a paradigm shift in understanding endometrial stem cell dysregulation in this condition. Our findings demonstrate that NUMB upregulation is not merely an epiphenomenon but rather a functionally relevant alteration with direct implications for disease pathogenesis and patient management.

The observed 3.2-fold increase in NUMB expression in adenomyotic tissues compared to controls (*p* < 0.001) establishes NUMB as a potential diagnostic biomarker for adenomyosis. The correlation between NUMB expression levels and clinical symptom severity provides an objective measure of disease activity, which is particularly valuable given the current limitations in adenomyosis diagnosis that rely heavily on imaging modalities with variable sensitivity and specificity.

Regarding the potential of NUMB as a therapeutic target specific to adenomyosis, several considerations support this possibility despite its presence in normal endometrium and myometrium. First, the observed elevated expression levels of NUMB in adenomyosis tissues (2–3-fold higher than controls) establishes NUMB as a potential diagnostic biomarker for adenomyosis. The correlation between NUMB expression levels and clinical symptom severity suggests that NUMB quantification could serve as an objective measure of disease activity. This is valuable given the current limitations in adenomyosis diagnosis, which relies heavily on imaging modalities with variable sensitivity and specificity.

The predominant single-cell distribution pattern of NUMB-positive cells, rather than clustered expression, indicates a diffuse alteration in cellular behavior throughout the adenomyotic tissue. This pattern suggests that NUMB dysregulation affects individual cell fate decisions rather than representing clonal expansion, supporting the stem cell dysregulation hypothesis in adenomyosis pathogenesis.

Our comprehensive bioinformatics analysis establishes NUMB as a central hub protein in endometrial pathology networks. The STRING network analysis revealed NUMB’s role as a key regulatory protein with 20 high-confidence interactions, particularly enriched in Notch signaling pathway components (FDR = 1.0 × 10^−15^). This systems-level approach demonstrates that NUMB alterations have implications extending beyond adenomyosis to endometrial cancer prognosis and survival outcomes.

The integration of tissue-based expression analysis with large-scale genomic data (TCGA) provides compelling evidence for NUMB’s central role in endometrial pathology. The identification of NUMB as a prognostic biomarker in endometrial cancer (HR = 0.42, 95% CI: 0.22–0.81) suggests that adenomyosis-associated NUMB alterations may confer protective effects against malignant transformation, which has immediate clinical relevance for risk stratification and surveillance strategies.

Therapeutic targeting of NUMB presents both opportunities and challenges. While complete NUMB inhibition could disrupt physiological endometrial regeneration and menstrual cycling, therapeutic strategies could aim to modulate rather than inhibit the pathological overexpression of NUMB. This approach could potentially target dysregulated isoforms or downstream effectors while preserving normal stem cell function.

The existence of multiple NUMB isoforms generated through alternative splicing offers potential for selective therapeutic targeting. The long isoform (NUMB-L) is primarily expressed in epithelial cells, where it regulates cell polarity and differentiation, while the short isoform (NUMB-S) predominates in stromal cells and modulates cell migration and invasion. Understanding isoform-specific expression patterns in adenomyosis could enable more precise therapeutic interventions.

Several limitations should be acknowledged in our study. The relatively small sample size, particularly for secretory phase analysis, limits the generalizability of our findings. Additionally, the whole-tissue analysis approach without separation into basal and functional endometrial layers could be addressed through emerging spatial transcriptomic and single-cell RNA sequencing approaches. These technologies would enable precise localization of NUMB expression patterns and identification of specific cell populations driving the observed alterations.

Future studies should focus on functional validation of NUMB’s role in adenomyosis pathogenesis through in vitro and in vivo models. Additionally, longitudinal studies examining NUMB expression changes during disease progression could provide insights into its utility as a monitoring biomarker. The development of NUMB-targeted therapeutic approaches requires careful consideration of tissue-specific effects and potential impact on normal endometrial function.

The clinical translation potential of our findings is substantial. NUMB represents a promising diagnostic and prognostic biomarker that bridges benign adenomyosis and malignant endometrial transformation. The correlation with symptom severity suggests potential utility in disease monitoring and treatment response assessment. Furthermore, the identification of NUMB as a central hub in endometrial pathology networks provides a foundation for developing targeted therapeutic strategies.

Our study provides the first molecular characterization of NUMB in adenomyosis, filling a critical gap in understanding stem cell regulatory mechanisms in this condition. The integration of experimental findings with computational analysis establishes a robust framework for future research and clinical applications in endometrial pathology.

## 5. Conclusions

This study presents the first evidence of altered NUMB expression in adenomyosis, demonstrating significant upregulation in both eutopic endometrium and myometrium compared to controls. The comprehensive bioinformatics analyses establish NUMB as a central hub protein in endometrial pathology, with significant prognostic value in endometrial cancer. NUMB represents a promising diagnostic and prognostic biomarker that bridges benign adenomyosis and malignant endometrial transformation, offering immediate translational potential for clinical risk stratification and therapeutic target development**.** These findings suggest that NUMB dysregulation may serve as a biomarker for endometrial stem cell dysfunction in adenomyosis and provide mechanistic insights into invasive endometrial diseases. Future research should focus on functional validation and therapeutic target development based on NUMB pathway modulation.

## Figures and Tables

**Table 1:** Clinical characteristics and symptomatology of adenomyosis and control groups.

Variable	Adenomyosis Group (*n* = 21)	Control Group (*n* = 14)
Age (years)	42.1 ± 6.2	43.4 ± 9.1
Gravidity (G)	1.9 ± 2.1	1.8 ± 1.6
Parity (P)	1.3 ± 1.5	1.2 ± 1.3
Cycle Phase		
Proliferative	17 (81.0%)	11 (78.6%)
Secretory	3 (14.3%)	3 (21.4%)
Atrophic	1 (4.8%)	0 (0%)
Symptoms		
Dysmenorrhea	12 (57.1%)	6 (42.9%)
Hypermenorrhea	8 (38.1%)	5 (35.7%)
Dyspareunia	7 (33.3%)	4 (28.6%)
Dysuria	6 (28.6%)	3 (21.4%)
Dyschezia	4 (19.0%)	3 (21.4%)
Chronic abdominal pain	7 (33.3%)	2 (14.3%)
Menometrorrhagia	3 (14.3%)	4 (28.6%)
Anemia	2 (9.5%)	0 (0%)
Premenstrual syndrome	1 (4.8%)	0 (0%)
CIN III	0 (0%)	1 (7.1%)

## Data Availability

The original contributions presented in this study are included in the article/Supplementary Materials. Further inquiries can be directed to the corresponding author. Protein interaction data is publicly available through the STRING database (https://string-db.org/). TCGA expression data is available through the TCGA Data Portal (https://www.cancer.gov/tcga (accessed on 2 November 2025)).

## Supplementary Materials

The following supporting information can be downloaded at: https://www.mdpi.com/article/10.3390/cimb47121027/s1.

## Abbreviations

The following abbreviations are used in this manuscript:

ESP	endometrial side population
DSL	Delta/Serrate/LAG-2
TP53	tumor protein p53
E3-ligase Mdm2	E3 ubiquitin-protein ligase Mouse double minute 2 homolog
PTB	phosphotyrosine-binding domain
IRB	Institutional Review Board
PBS	phosphate-buffered saline
BSA	bovine serum albumin
SD	standard deviation
