# Supplementary material for: NUMB in Endometrial Pathology: From Adenomyosis Expression Patterns to Endometrial Cancer Survival Implications
Source: Curr Issues Mol Biol. 2025 Dec 10;47(12):1027. doi: 10.3390/cimb47121027 (PMC12731609; doi:10.3390/cimb47121027)
Supplement: Supplementary file 1 [file cimb-47-01027-s001.zip › cimb-3999545-supplementary.pdf]

Table S1. Analysis of NUMB Alterations in Endometrial Carcinoma. The Cancer Genome Atlas (TCGA) Uterine Corpus Endometrial Carcinoma (UCEC) dataset, 560 patients with complete genomic and clinical data, cBioPortal for Cancer Genomics (www.cbioportal.org), reference genome: GRCh37/hg19. Fisher's exact test for categorical comparisons, Mann–Whitney U test for continuous variables, Kaplan–Meier method for survival analysis, Log-rank test for survival comparisons, and multiple testing correction: Benjamini–Hochberg FDR.

A. Overall Alteration Frequency

**Alteration Type**	**Number of Cases**	**Frequency (%)**	**95% CI**	**Clinical Significance**
Total Alterations	69/560	12.3	9.7-15.3	Moderate frequency
Amplifications	38/560	6.8	4.9-9.2	Most common alteration
Deep Deletions	12/560	2.1	1.1-3.7	Rare but significant
Missense Mutations	16/560	2.8	1.6-4.6	Functional impact likely
Truncating Mutations	3/560	0.6	0.1-1.6	Severe functional loss

B. Mutation Subtype Details

**Mutation Category**	**Specific Alteration**	**Count**	**Frequency (%)**	**Protein Impact**	**Functional Consequence**
**Amplifications**					
	High-level amplification (>4 copies)	15	2.7	Overexpression	Enhanced Notch inhibition
	Moderate amplification (3-4 copies)	23	4.1	Increased expression	Moderate Notch suppression
**Deletions**					
	Homozygous deletion	4	0.7	Complete loss	Loss of Notch regulation
	Heterozygous deletion	8	1.4	Reduced expression	Partial Notch dysregulation
**Point Mutations**					
	PTB domain mutations	8	1.4	Domain disruption	Impaired protein binding
	PRR domain mutations	5	0.9	Regulatory disruption	Altered phosphorylation
	Other missense mutations	3	0.5	Variable impact	Context-dependent
**Truncating Mutations**					
	Nonsense mutations	2	0.4	Premature termination	Complete functional loss
	Frameshift mutations	1	0.2	Reading frame disruption	Nonfunctional protein

C. Histological Subtype Distribution

**Histological Subtype**	**Total Cases**	**NUMB Alterations**	**Frequency (%)**	***p*-value***	**Odds Ratio (95% CI)**
**Endometrioid**	417	45	10.8	Reference	1.00

**Serous**	95	18	18.9	0.032	1.92 (1.06-3.47)
**Mixed**	28	4	14.3	0.542	1.37 (0.46-4.08)
**Clear Cell**	20	2	10.0	0.999	0.92 (0.21-4.05)

D. Molecular Subtype Distribution

**Molecular Subtype**	**Total Cases**	**NUMB Alterations**	**Frequency (%)**	***p*-value***	**Clinical Relevance**
**POLE Ultramutated**	65	12	18.5	0.045	High mutation burden
**MSI Hypermutated**	158	22	13.9	0.387	Moderate frequency
**Copy Number Low**	231	21	9.1	0.089	Lower alteration rate
**Copy Number High**	106	14	13.2	0.756	Chromosomal instability

E. Co-occurring Alterations

**Gene**	**Co-alteration Frequency**	***p*-value***	**Biological Relationship**
**PTEN**	34.8%	0.012	PI3K/AKT pathway interaction
**PIK3CA**	28.9%	0.045	Pathway convergence
**CTNNB1**	23.2%	0.078	Wnt signaling crosstalk
**TP53**	21.7%	0.156	Cell cycle regulation
**KRAS**	18.8%	0.234	Growth factor signaling
